# Repression of *FLOWERING LOCUS T* Chromatin by Functionally Redundant Histone H3 Lysine 4 Demethylases in *Arabidopsis*

**DOI:** 10.1371/journal.pone.0008033

**Published:** 2009-11-25

**Authors:** Ju-Hee Jeong, Hae-Ryong Song, Jong-Hyun Ko, Young-Min Jeong, Young Eun Kwon, Jae Hong Seol, Richard M. Amasino, Bosl Noh, Yoo-Sun Noh

**Affiliations:** 1 School of Biological Sciences, Seoul National University, Seoul, Korea; 2 Global Research Laboratory for Floral Regulatory Signaling, Seoul National University, Seoul, Korea; 3 Environmental Biotechnology National Core Research Center, Gyeongsang National University, Jinju, Korea; 4 Department of Biochemistry, University of Wisconsin, Madison, Wisconsin, United States of America; Ecole Normale Superieure, France

## Abstract

FLOWERING LOCUS T (FT) plays a key role as a mobile floral induction signal that initiates the floral transition. Therefore, precise control of *FT* expression is critical for the reproductive success of flowering plants. Coexistence of bivalent histone H3 lysine 27 trimethylation (H3K27me3) and H3K4me3 marks at the *FT* locus and the role of H3K27me3 as a strong *FT* repression mechanism in *Arabidopsis* have been reported. However, the role of an active mark, H3K4me3, in *FT* regulation has not been addressed, nor have the components affecting this mark been identified. Mutations in *Arabidopsis thaliana Jumonji4* (*AtJmj4*) and *EARLY FLOWERING6* (*ELF6*), two *Arabidopsis* genes encoding Jumonji (Jmj) family proteins, caused *FT*-dependent, additive early flowering correlated with increased expression of *FT* mRNA and increased H3K4me3 levels within *FT* chromatin. Purified recombinant AtJmj4 protein possesses specific demethylase activity for mono-, di-, and trimethylated H3K4. Tagged AtJmj4 and ELF6 proteins associate directly with the *FT* transcription initiation region, a region where the H3K4me3 levels were increased most significantly in the mutants. Thus, our study demonstrates the roles of AtJmj4 and ELF6 as H3K4 demethylases directly repressing *FT* chromatin and preventing precocious flowering in *Arabidopsis*.

## Introduction

Flowering, a critical developmental transition in plants, is controlled by both environmental cues and internal developmental signals. Photoperiod exerts profound effects on flowering in numerous plant species including Arabidopsis. Generation of the photoperiodic floral induction signal in the leaves is mediated by light and circadian-clock signaling and relayed through the photoperiod pathway. *GIGANTEA* (*GI*) [1], [2] and *CONSTANS* (*CO*) [3] act as upstream activators of *FLOWERING LOCUS T* (*FT*) [4], [5] in the photoperiod pathway. On the other hand, *FT* expression is repressed by *FLOWERING LOCUS C* (*FLC*) [1], [2], and this repression is mediated possibly by a protein complex between FLC and SHORT VEGETATIVE PHASE [6]. Thus, *FT* acts not only as a component in the photoperiod pathway but also as a floral integrator that combines the perception of inductive photoperiods and the *FLC*-mediated floral repression signal. FT protein, as a graft-transmissible signal, is translocated from the vascular tissue of leaves to the shoot apex [7], where it interacts with FD and stimulates the floral transition [8], [9].

Recent studies have shown that *FT* expression is affected by histone modifications. The *FT* locus was shown to be enriched with trimethylated histone H3 lysine 27 (H3K27me3) [10], [11], and loss of putative Polycomb Repressive Complex 2 (PRC2) components results in decreased H3K27me3 within *FT* chromatin, which in turn increases *FT* expression [12]. Furthermore, lack of LIKE-HETEROCHROMATIN PROTEIN1 (LHP1), which can bind to H3K27me3 and silence chromatin [10], [11], also causes increased *FT* expression [13], [14]. Therefore, *FT* transcription is repressed by H3K27me3 and its effector protein (LHP1).

Methylation at histone residues can contribute to mitotically stable epigenetic changes in gene expression. In contrast it has recently been demonstrated that at least two classes of enzymes are capable of removing methyl groups from either histone lysine or arginine (R) residues and potentially reversing epigenetic changes in gene expression. Human Lysine-Specific Demethylase1 (LSD1), a nuclear amine oxidase, specifically demethylates mono- and dimethylated but not trimethylated H3K4 [15]. After the discovery of LSD1, a human Jmj C domain-containing protein, JHDM1A, was first shown to be able to remove methyl groups from H3K36 [16]. Soon after the identification of JHDM1A, a number of JmjC domain-containing proteins have been demonstrated to be H3K4, H3K9, H3K27, H3K36, H3R2, and H4R3 demethylases [17]–[19]. Unlike LSD1, JmjC domain-containing proteins are capable of demethylating all of the mono-, di- and trimethylated lysines of histones [20]. Thus, JmjC family proteins are considered as the major histone demethylases in eukaryotic cells.

Arabidopsis has twenty-one genes encoding JmjC family proteins (*Arabidopsis thaliana Jumonji* (*AtJmj*) *1∼21*) [21]. To date, three of these genes have been functionally characterized. *EARLY FLOWERING6* (*ELF6*; *AtJmj1*) and *RELATIVE OF EARLY FLOWERING6* (*REF6*; *AtJmj2*) were shown to be involved in photoperiodic flowering and *FLC* regulation, respectively [22]. INCREASED EXPRESSION OF BONSAI METHYLATION 1 (IBM1; AtJmj15), represses genic cytosine methylation, possibly through demethylation of H3K9me [23]. In this report, we show that ELF6 and another Arabidopsis JmjC family protein (AtJmj4) directly repress *FT* expression via demethylation of H3K4me. Thus, our study demonstrates the presence of an H3K4me demethylation-mediated mechanism in addition to the previously characterized H3K27 methylation-mediated mechanism in the chromatin repression of a key flowering time regulator, *FT*.

## Results

### Mutations in *AtJmj4* Cause Early Flowering

**Figure 1:**
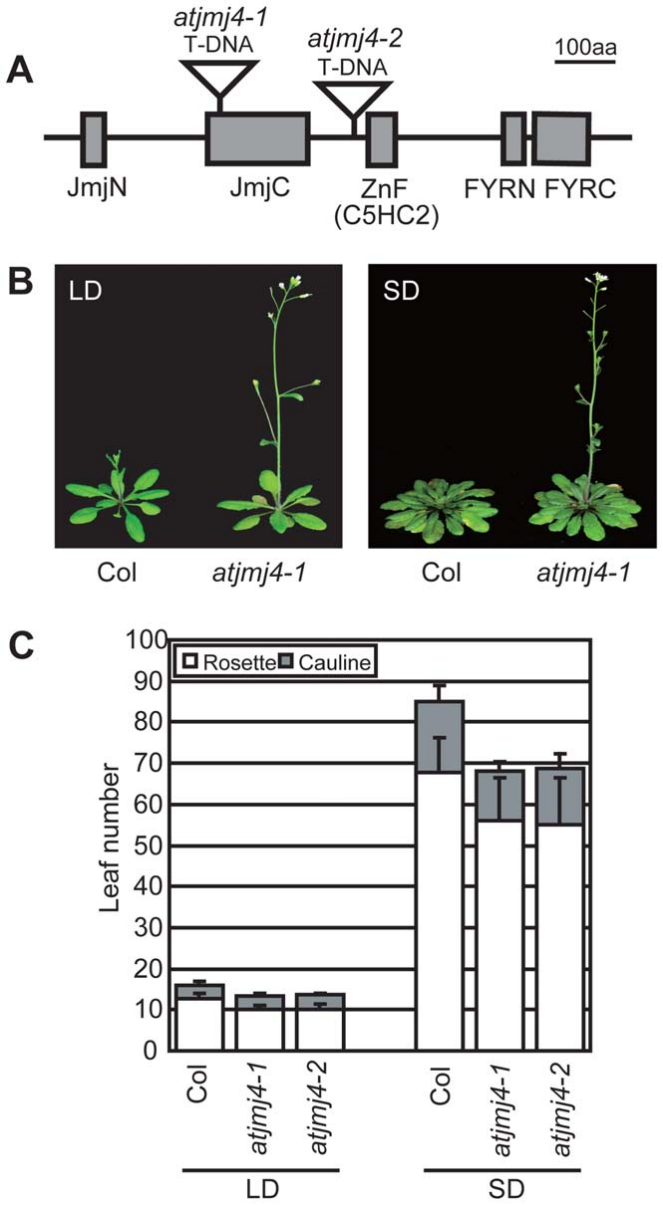
Early flowering of *atjmj4* mutants. A) Domain organization of AtJmj4. Domains were predicted by SMART (http://smart.embl-heidelberg.de/). Lines indicate interdomain regions. T-DNA insertion sites on the genomic sequence of *AtJmj4* in *atjmj4-1* and *atjmj4-2* are marked on the corresponding positions of their translated protein products. B) Early flowering phenotype of *atjmj4-1* mutant plants grown in either LD or SD. C) Flowering time of *atjmj4* mutants. Wt Col and *atjmj4* mutant plants were grown in either LD or SD and their flowering times were determined as the number of primary rosette and cauline leaves formed at bolting. At least 12 individuals were scored for each genotype. Error bars represent sd.

To address the biological roles of Arabidopsis JmjC domain-containing proteins, we obtained T-DNA insertion lines of the corresponding genes from the SALK T-DNA collection and evaluated their phenotypes. Two independent homozygous T-DNA insertion mutants of *Arabidopsis thaliana Jumonji4* (*AtJmj4* or *At4g20400*) [21] showed an early flowering phenotype both in long days (LD; 16 h light/8 h dark) and short days (SD; 8 h light/16 h dark; **Figures 1A–1C**). The early flowering phenotype was not due to an accelerated leaf initiation rate (**Figure S1**) but resulted from a more rapid developmental transition of the shoot apical meristem (SAM) from the vegetative to the reproductive phase as characterized by a lower number of rosette and cauline leaves at the onset of flowering (**Figure 1C**). No other visible phenotypic traits were apparent in *atjmj4* mutants. Plants heterozygous for the T-DNA insertions displayed a wild-type (wt) flowering time (data not shown), indicating that *atjmj4-1* and *atjmj4-2* are fully recessive mutations with respect to flowering time. Because both alleles displayed similar early flowering behaviors, *atjmj4-1* was selected for the genetic and molecular analyses we report herein.

### Early Flowering of *atjmj4* Is Due to Increased Expression of *FT*

**Figure 2:**
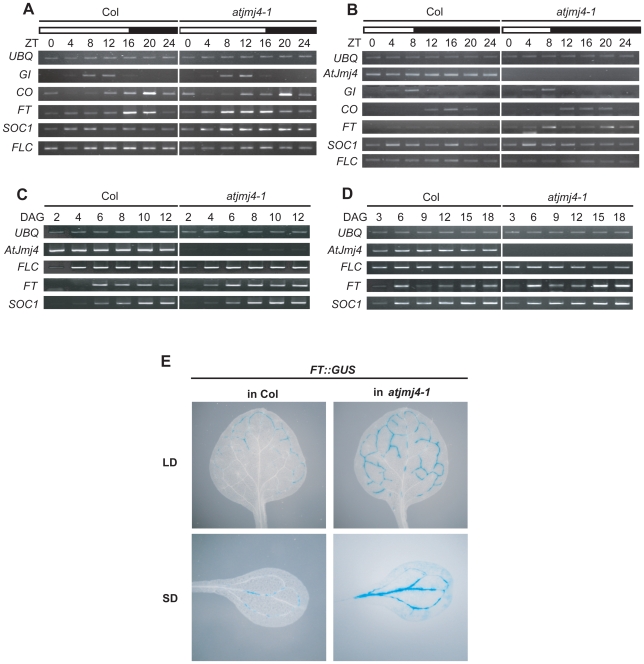
Increased expression of *FT* in *atjmj4* mutants. A and B) Expression of flowering genes in *atjmj4-1* mutants. Col and *atjmj4-1* plants were grown in LD (A) for 10 days (d) or in SD (B) for 15 d, harvested every 4 hours (h) at indicated zeitgeber (ZT; h after light-on) for one d, and used for RT-PCR analyses. *Ubiquitin* (*UBQ*) was included as an expression control. Identical results were obtained from two independent experiments, and one of them is shown. White and black bars represent light and dark periods, respectively. C and D) Temporal expression of flowering genes in *atjmj4-1* mutants. Col and *atjmj4-1* plants were grown for up to 12 days after germination (DAG) in LD (C) or 18 DAG in SD (D). Plants were harvested during the growth period at ZT14 (LD) or ZT8 (SD) of designated DAG and used for RT-PCR analyses because *FT* mRNA expression peaked at the ZT in each photoperiod. E) Histochemical GUS staining of transgenic plants harboring *FT::GUS* fusion construct in Col or *atjmj4-1* plants. Plants were grown for 16 d either in LD or SD before GUS staining.

Because of the early-flowering phenotype of loss of AtJmj4, we evaluated whether it might have a role in the expression of key flowering time regulators, such as *GI*, *CO*, *FT*, *FLC*, and *SUPPRESSOR OF OVEREXPRESSION OF CO1* (*SOC1*), a floral integrator [24], [25] (**Figure 2**). In LD, the mRNA levels of *GI* and *CO* were not affected by the *atjmj4-1* mutation. However, the mRNA levels of the floral integrators *FT* and *SOC1* were up-regulated in *atjmj4-1* and that of *FLC* was slightly reduced (**Figure 2A**). To further explore these observations, we monitored the expression of these genes in SD (**Figure 2B**). *FT* mRNA levels were consistently higher in the *atjmj4-1* mutants in SD, but the expression of *SOC1* mRNA was not affected significantly. Because *FT* induction by *CO* requires the stabilization of CO protein by light, which occurs only in LD in Arabidopsis, the up-regulation of *FT* by the *atjmj4-1* mutation in SD might be caused by de-repression instead of induction. The mRNA level of *FLC* was slightly reduced in the *atjmj4-1* mutants in SD compared to wt as was the case in LD.

We then compared the mRNA levels of *FLC*, *FT*, and *SOC1* in wt versus *atjmj4-1* at different developmental stages both in LD and SD (**Figures 2C** and **2D**). Notably, *FT* mRNA levels were higher in *atjmj4-1* than in wt throughout the developmental stages tested. Moreover, the *FT* promoter showed increased activity both in LD and SD in the *atjmj4-1* homozygous mutants when the construct containing the *FT* promoter fused with *β*-*glucuronidase* (*FT::GUS*) [14] was compared to expression levels in wt Col (**Figure 2E**). *FT::GUS* expression which was detected in the marginal minor veins of wt leaves was also observed in the central minor veins of *atjmj4-1* mutant leaves in LD. In SD, *FT::GUS* expression was robust in the major veins of *atjmj4-1* mutant leaves, although its expression was weak in the same wt leaf tissue. Thus, the *atjmj4* mutation leads to increased expression of *FT* mRNA through enhanced activity of *FT* promoter. However, the cell-type specific expression pattern of *FT* in leaf veins was not affected by the *atjmj4* mutation.

**Figure 3:**
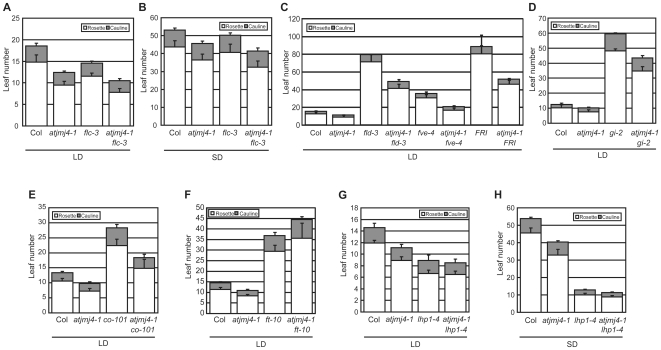
Genetic interaction between *Atjmj4* and other flowering time genes. A and B) Flowering time of *atjmj4-1 flc-3* double mutants. C) Genetic interaction between *AtJmj4* and *FLC* regulators. D to F) Genetic interaction between *AtJmj4* and photoperiod-pathway genes. G and H) Genetic interaction between *AtJmj4* and *LHP1*. Flowering times were determined in LD (A and C to G) or SD (B and H). At least 12 individuals were scored for each genotype (A to H). Error bars represent sd (A to H).

Because the *atjmj4* mutation also caused a slight reduction in *FLC* mRNA levels (**Figures 2A–2D**), we tested whether the decrease in *FLC* expression was a cause for the increased expression of *FT* in the mutants. For this, we first compared *FT* mRNA levels between an *flc* null mutant (*flc-3*) [26] and *atjmj4-1* (**Figure S2**). Although the expression levels of *GI*, *CO*, and *SOC1* mRNAs were similar in the two genotypes, *FT* mRNA level was clearly higher in *atjmj4-1*. Furthermore, when we compared the flowering times between *atjmj4-1* single and *atjmj4-1 flc-3* double mutants both in LD and SD, the double mutants flowered earlier than the single mutants in both photoperiodic conditions (**Figures 3A** and **3B**). Because vernalization can reduce *FLC* expression and *VERNALIZATION INSENSITIVE3* (*VIN3*) is essential for the process [27]–[29], we tested a possibility of constitutive vernalization response in *atjmj4* using *atjmj4* single and *atjmj4 vin3* double mutants. *atjmj4* showed a normal response to vernalization, whereas *atjmj4 vin3* did not (**Figure S3**). Further, *atjmj4 vin3* flowered earlier than *vin3* without or with vernalization (**Figure S3**). Therefore, data above altogether indicate that the *atjmj4* mutation causes an early flowering independently of *FLC* expression.

That AtJmj4 acted downstream of *FLC* was reinforced by our studies on genetic interactions between *atjmj4* and several autonomous-pathway mutants [30] (**Figure 3C**). Double mutants between *atjmj4* and *flowering locus d* (*fld*) [31] or *fve* [32] exhibited flowering times that were intermediate relative to each single mutant. When a functional *FRIGIDA* (*FRI*) [33], [34] allele was introduced into *atjmj4*, *atjmj4-1 FRI* also flowered at intermediate time between *atjmj4-1* and *FRI*. These results indicate that AtJmj4 controls flowering mainly through an *FLC*-independent pathway.

To test interactions between *AtJmj4* and genes acting in the photoperiod pathway the following double mutants were analyzed: *atjmj4-1 gi-2*, *atjmj4-1 co-101*, and *atjmj4-1 ft-10*. The early-flowering phenotype of *atjmj4-1* was attenuated by the LD-specific late flowering phenotypes of *gi-2* and *co-101* (**Figures 3D** and **3E**). However, the early flowering of *atjmj4-1* was fully suppressed by the *ft-10* mutation (**Figure 3F**). Therefore, AtJmj4 might affect *FT* expression independently of GI and CO. Consistent with this hypothesis, *FT* mRNA level in *atjmj4-1 gi-2* or *atjmj4-1 co-101* double mutants was higher compared to that in *gi-2* or *co-101* single mutants, respectively (**Figure S4**). LHP1 directly represses *FT* chromatin [10], [11] such that *FT* is strongly de-repressed in *lhp1* mutants [13]. Consistent with the repressive roles of AtJmj4 and LHP1 in *FT* expression, *atjmj4-1 lhp1-4* double mutants flowered at similar times with the severe early flowering mutant *lhp1-4* in both LD and SD (**Figures 3G** and **3H**).

The data above indicated that AtJmj4 acts as an *FT* repressor, and thus it was of interest to test if AtJmj4 affects *FT* expression indirectly through controlling the expression of *FT* regulators. To address this, we compared the mRNA levels of known *FT* regulators, namely *TARGET OF EAT1* (*TOE1*), *TOE2*, *TOE3* [35], [36], *SCHLAFMÜTZE* (*SMZ*), *SCHNARCHZAPFEN* (*SNZ*) [35], [37], *CRYPTOCHROME-INTERACTING BASIC-HELIX-LOOP-HELIX1* (*CIB1*) [38], *TEMPRANILLO1* (*TEM1*), *TEM2* [39], *SHORT VEGETATIVE PHASE* (*SVP*) [6], [40], [41], *AGAMOUS-like15* (*AGL15*), and *AGL18* [42], between wt and *atjmj4* mutants, but none showed detectable differences (**Figure S5**).

### AtJm4 Is a Nuclear Protein Preferentially Expressed in Vascular Tissues and Shoot/Root Apices

**Figure 4:**
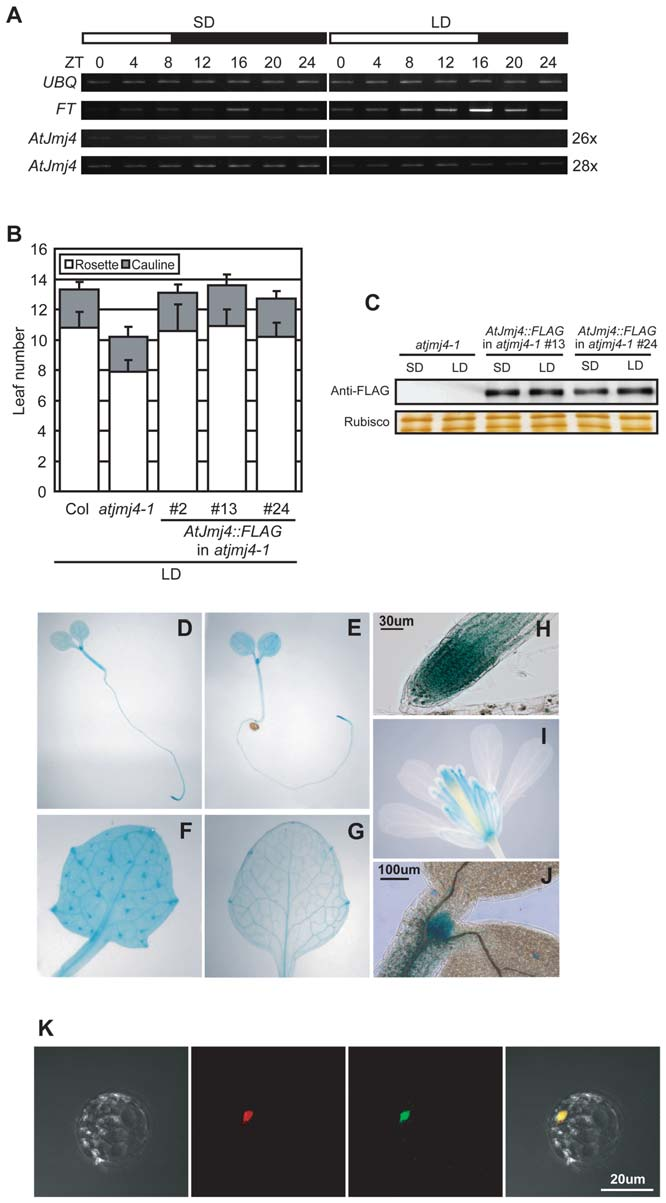
*AtJmj4* expression. A) mRNA expression of *AtJmj4* in SD and LD. Wt Col plants were grown in SD for 12 d or in LD for 8 d and used for RT-PCR analyses. *UBQ* was used as an expression control. Number of PCR cycles used for *AtJmj4* is indicated on the right. B) Genomic complementation of *atjmj4-1*. Three independent transgenic lines of *atjmj4-1* containing *AtJmj4::FLAG* (see text for details) were grown in LD and their flowering times were determined as the number of rosette and cauline leaves formed at bolting. At least 12 individuals were scored for each genotype. Error bars represent sd. C) Expression of the AtJmj4::FLAG fusion protein in SD and LD. Plants of *atjmj4-1* and two of the complementation lines shown in (B) were grown for 12 d in SD or 8 d in LD, harvested at ZT12, and used for Western blot analyses. Upper panel: Western blot with anti-FLAG antibody. Lower panel: Silver stained gel image of rubisco subunits. D to J) Histochemical GUS staining of transgenic Col plants harboring *AtJmj4::GUS*. Plants grown in LD (D and I) or SD (E, F, G, H, and J) were used for GUS staining. (D) In 4 d-old seedling. (E) In 6-d old seedling. (F) In trichomes. (G) In leaf. (H) In root tip. (I) In floral organs. (J) In shoot apex. K) Subcellular localization of AtJmj4 in Arabidopsis mesophyll protoplast. From left to right; bright-field image, LHP1::RFP fusion protein, AtJmj4::GFP fusion protein, merged image of the left three images.

When we compared the mRNA levels of *AtJmj4* between SD- and LD-grown seedlings at a similar developmental stage, we could observe higher expression of *AtJmj4* mRNA in SD-grown seedlings, although the *FT* mRNA levels were clearly higher in LD-grown seedlings (**Figure 4A**). This observation was consistent with the higher *AtJmj4* promoter activity in SD than in LD as studied by using an *AtJmj4 promoter::GUS* fusion construct [21]. This might indicate a preferential repressive role of AtJmj4 in *FT* expression in SD, and thus we further evaluated whether the expression level of AtJmj4 protein was also higher in SD than in LD. For this, we made an *AtJmj4::FLAG* fusion construct that contained an *AtJmj4* promoter fragment, 3 copies of the FLAG tag, and the *AtJmj4* cDNA with the entire coding sequence. The construct fully rescued the early-flowering phenotype of *atjmj4-1* when introduced into the mutants (**Figure 4B**). When we measured the expression levels of the AtJmj4::FLAG fusion protein in transgenic plants at the same developmental stage with the one used to study the expression of *AtJmj4* mRNA (**Figure 4A**), however, there was no difference in the expression level between SD- and LD-grown seedlings (**Figure 4C**). Thus, AtJmj4 expression might be further controlled at posttranscriptional level(s), although its promoter activity *per se* is affected by day-length, and AtJmj4 protein exerts its repressive role for *FT* in both LD and SD.

Because AtJmj4 protein levels are constant at the whole plant level despite day-length dependent mRNA levels, we studied the spatial expression pattern of AtJmj4 protein using a construct harboring the entire genomic region of *AtJmj4* including 1.5 kb promoter in frame with *GUS* (*AtJmj4::GUS*). In seedlings, AtJmj4::GUS expression pattern was similar in SD and LD (**Figures 4D** and **4E**). The GUS activity was detected in most organs, but strong activity was observed in the shoot apex (**Figure 4J**), primary root tip (**Figure 4H**), trichomes of young leaves (**Figure 4F**), and leaf vascular tissues (**Figure 4G**). In floral organs, strong GUS activity was detected in anther filaments and styles (**Figure 4I**). Importantly, the *AtJmj4::GUS* expression domain showed an overlap with the *FT* expression domain [14] in leaves.

The subcellular localization of AtJmj4 protein was evaluated by a protoplast transfection assay using a fusion protein between AtJmj4 and green fluorescence protein (GFP; AtJmj4::GFP) expressed from the *Cauliflower Mosaic Virus 35S* (*CaMV35*) promoter. A fusion protein between LHP1 and red fluorescence protein (RFP; LHP1::RFP), which was known to be localized into the nucleus [43], was co-expressed with AtJmj4::GFP. Both RFP and GFP signals were detected only in the nucleus (**Figure 4K**). This result is in agreement with the possible role of AtJmj4 as a chromatin and/or transcriptional regulator.

### AtJmj4 and ELF6 Play Redundant Roles in *FT* Repression as H3K4-Specific Demethylases

**Figure 5:**
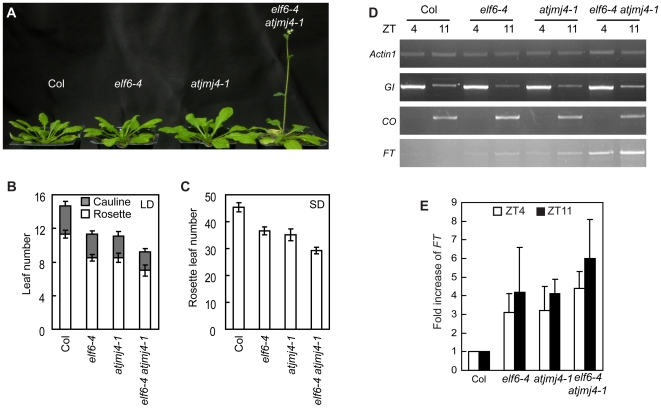
Additive effect of *elf6* and *atjmj4* mutations on *FT*-dependent early flowering. A) Early flowering phenotype of *elf6-4 atjmj4-1* double mutant. All plants were grown in SD for 63 d before taken picture. B and C) Flowering time of wt Col, *elf6-4*, *atjmj4-1*, and *elf6-4 atjmj4-1* double mutants in LD and SD as determined by number of leaves formed at bolting. At least 15 individuals were scored for each genotype. Error bars represent sd. D) Expression of flowering genes in *elf6-4 atjmj4* double mutants. Plants of each genotype were grown in SD for 57 d and harvested at ZT4 or ZT11 for RT-PCR analyses. *Actin1* was included as an expression control. Identical results were obtained from two independent experiments and one of them is shown. E) qPCR analysis of *FT* expression. The same RNAs used in (D) were evaluated. The wt Col levels were set to 1 after normalization by *Actin1* for qPCR analysis. Error bars represent sd.

In our previous study, we reported that *ELF6* (*At5g04240*), a gene encoding an Arabidopsis Jmj-domain protein, acts as a repressor in the photoperiodic flowering pathway [22]. Therefore, it was of interest to study the relationship between *AtJmj4* and *ELF6* in the regulation of photoperiodic flowering. For this, an *elf6-4 atjmj4-1* double mutant was generated and assayed for flowering time. The double-mutant plants flowered earlier than either single mutants as well as the wt Col plants both in LD and SD (**Figures 5A–5C**). Because both *ELF6* and *AtJmj4* have repressive roles in the photoperiod pathway, we then evaluated the mRNA levels of genes acting in the photoperiod pathway, namely *GI*, *CO* and *FT*, using RNAs isolated from SD-grown plants. mRNA levels of *GI* and *CO* were similar among wt, the *elf6-4* and *atjmj4-1* single mutants, and the *elf6-4 atjmj4* double mutants at ZT4 and ZT11 (**Figure 5D**). However, *FT* mRNA level was increased in the *elf6-4* and *atjmj4-1* single mutants by at least 3 fold compared to that in wt, and this increase was more significant in the *elf6-4 atjmj4-1* double mutants (**Figures 5D** and **5E**). Therefore, the data for *FT* mRNA expression as well as the flowering time (**Figures 5A–5C**) indicate that *ELF6* and *AtJmj4* have redundant repressive roles in photoperiodic flowering through negatively regulating *FT* expression.

**Figure 6:**
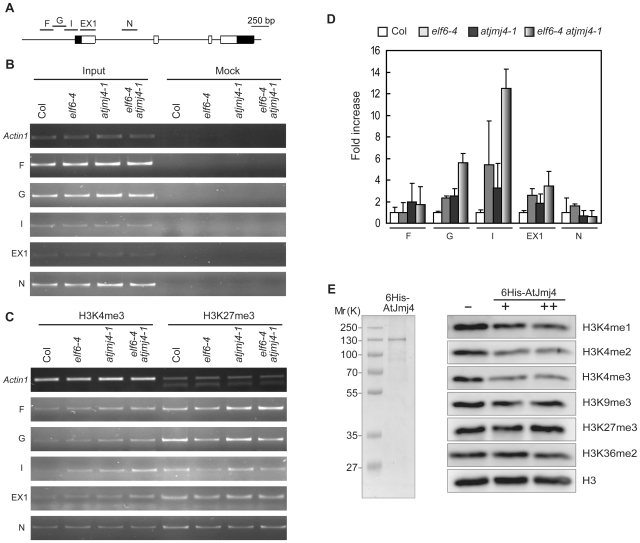
Increased trimethylation of H3K4 at *FT* locus by *elf6* and *atjmj4* mutations. A) Schematic of *FT* locus showing regions (F, G, I, EX1, and N) amplified by the primers used for ChIP analysis. The front and the rear black boxes indicate 5′ and 3′ UTRs, respectively. White boxes indicate exons, while lines indicate introns and intergenic regions. B and C) ChIP assay of *FT* chromatin with antibody against H3K4me3 or H3K27me3. Plants of each genotype were grown in SD for 57 d and harvested for ChIP assay. ‘Input’ indicates chromatins before immunoprecipitation. ‘Mock’ refers to control samples lacking antibody. *Actin1* was used as an internal control. D) qPCR analysis of the ChIP assay for H3K4me3 described in (B and C). The wt Col levels were set to 1 after normalization by input. Error bars represent sd. E) Coomassie-blue stained 6His-AtJmj4 protein purified from *sf9* cells (left), and *in vitro* histone demethylation activity assay using the purified protein (right). Assays were performed without (−) or with either two (+) or four (++) µg of purified 6His-AtJmj4 protein. Mr (K), molecular mass in kilo-daltons.

Our unpublished phylogenetic analysis of the JmjC domains of ELF6, AtJmj4, and human Jmj proteins showed that the JmjC domains of ELF6 and AtJmj4 are clustered along with the JmjC domains of human JARID1 family which is known to specifically demethylate H3K4me3 and H3K4me2 [17], [19]. Hence, we evaluated whether the level of H3K4me within *FT* chromatin is affected by *elf6* and *atjmj4* mutations using chromatin immunoprecipitation (ChIP) assays. Sets of primers covering different regions of *FT* locus were used (**Figure 6A**). H3K4me3 levels were increased in G, I, and EX1 regions of *FT* locus by the *elf6-4* and *atjmj4-1* mutations, and the increase was more significant when both the mutations were combined (**Figures 6B–6D**). However, H3K4me3 levels in regions F and N were not affected significantly by these mutations. The level of another histone methylation, H3K27me3, which was reported to be enriched within *FT* chromatin [10], [11], was slightly reduced by the *elf6-4* but not by the *atjmj4-1* mutation in some of the *FT* regions tested (**Figure 6C**). These results indicate that ELF6 and AtJmj4 repress *FT* expression by negatively affecting the methylation of H3K4 but not H3K27 within *FT* chromatin.

To test if ELF6 and AtJmj4 are active histone demethylases, we tried to express the full-length ELF6 and AtJmj4 proteins in several expression systems. Although we could not express the full-length ELF6 in any systems employed, we could express the full-length AtJmj4 in insect *sf9* cells as an amino-terminal 6 histidine-tagged protein (6His-AtJmj4) which had a molecular mass of 130 kilo-daltons. 6His-AtJmj4 was purified to near homogeneity (**Figure 6E**) and subjected to *in vitro* histone demethylase activity assay. 6His-AtJmj4-mediated histone demethylase activity was analyzed by decreased signals in western blots with antibodies specific to methylated histone H3 residues (**Figure 6E**). Incubation of the recombinant 6His-AtJmj4 protein with histone substrates in the demethylase assays resulted in reduced levels of H3K4me1, H3K4me2, H3K4me3, but not of H3K9me3, H3K36me2, and H3K27me3 (**Figure 6E**). The levels of H3K4me3 and H3K4me2 were decreased more than the level of H3K4me1. These results indicate that AtJmj4 is an intrinsic H3K4-specific demethylase which has higher activity for H3K4me3 and H3K4me2 than H3K4me1.

**Figure 7:**
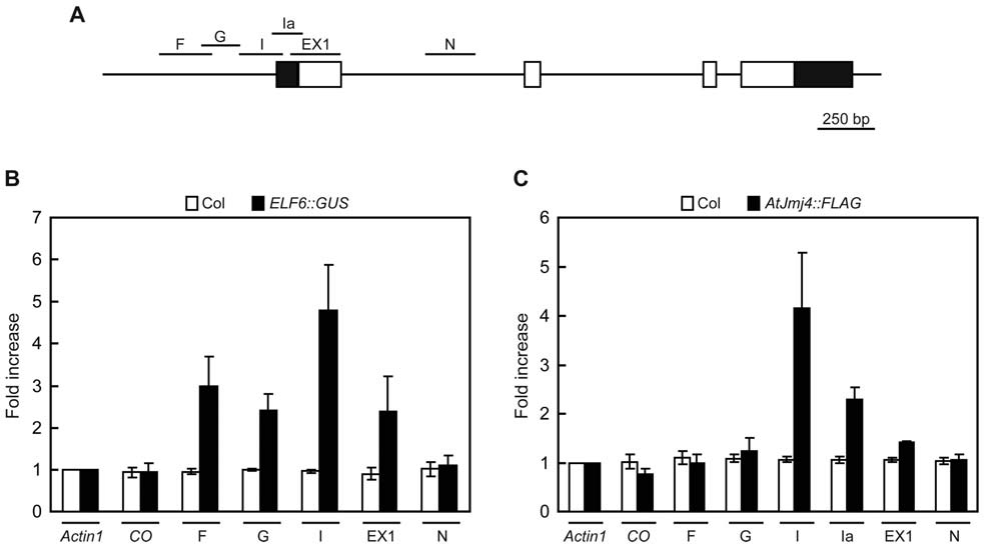
Direct association of ELF6 and AtJmj4 with *FT* chromatin. A) *FT* regions tested for ChIP assay. Schematic is as described in Figure 6A except for the region Ia, which was added in assays in (C). B) ELF6 binding to *FT* chromatin. LD grown 16 d-old wt Col and *ELF6::GUS*–containing transgenic *elf6-4* plants [22] were harvested and used for ChIP assay using GUS-specific antibody. Amount of immunoprecipitated chromatin was measured by qPCR (B and C). *Actin1* and *CO* were used as internal controls, and the level of *Actin1* in each sample was set to 1 for normalization (B and C). Error bars represent se of three independent biological replicates (B and C). C) AtJmj4 binding to *FT* chromatin. LD grown 16 d-old wt Col and *AtJmj4::FLAG*–containing *atjmj4-1* plants were harvested and used for ChIP assay using FLAG-specific antibody.

The results in **Figure 5** and **Figure 6** suggested that AtJmj4 and ELF6 might directly target *FT* chromatin and repress the transcription activity of *FT* by reducing the methylation level of H3K4. To test if *FT* chromatin is directly targeted by ELF6 and AtJmj4, we preformed ChIP assays using transgenic plants expressing functional *ELF6::GUS* [22] and *AtJmj4::FLAG* (**Figures 4B** and **4C**) as demonstrated by the complementation of the *elf6-4* and *atjmj4-1* mutant phenotypes, respectively. PCR was then carried out using primers amplifying various regions of *FT* locus (**Figure 7A**). ELF6::GUS showed binding to broad regions of the *FT* locus around the transcription start site with strongest binding to region I (**Figures 7B**). However, ELF6::GUS did not show binding to region N which is a part of the first intron of *FT* and the transcription initiation region (−117 to +71 from the transcription start site) of *CO*. AtJmj4::FLAG showed a similar binding pattern with ELF6::GUS to *FT* chromatin (**Figures 7C**). It showed strong bindings to regions I to EX1 like ELF6::GUS, but its binding to regions F and G was not detected unlike ELF6::GUS. AtJmj4::FLAG binding to region N and the transcription initiation region of *CO* was not detected as for the case of ELF6::GUS. In summary, both ELF6::GUS and AtJmj4::FLAG can associate directly and specifically with the transcription initiation region of *FT* locus where the H3K4me3 levels showed the largest increase in *elf6*, *atjmj4*, and *elf6 atjmj4* mutants (**Figure 6D**). Thus, ELF6 and AtJmj4 proteins directly target *FT* chromatin and regulate flowering time via demethylation of H3K4me.

## Discussion

Recent studies have shown that the expression of some key flowering genes, such as *FLC* and *FT*, are regulated through chromatin modifications and have also identified many of the factors involved in the chromatin modification processes [44], [45]. In this study, we show that AtJmj4 and ELF6 play a role in the repression of *FT* transcription by removing methyl groups from H3K4 at the *FT* locus.

*FT* chromatin contains bivalent marks; the active mark (H3K4me3) and the repressive mark (H3K27me3) exist simultaneously [12]. Of these, only H3K27me3 has been studied. It plays a critical role in preventing precocious floral transition by establishing and maintaining repressive *FT* chromatin as default state. The PRC2-like complex comprised of CURLY LEAF, SWINGER, EMBRYONIC FLOWER2, and FERTILIZATION INDEPENDENT ENDOSPERM, is required for H3K27me and the repression of *FT* [12].

Unlike H3K27me3, H3K4me3 has been known to be positively associated with transcriptional activity [46]. H3K4me3 can be recognized by the TFIID complex via the PHD finger of TAF3, which in turn recruits RNA polymerase II, leading to transcription activation [47]. In this study, we demonstrate that AtJmj4 is involved in *FT* repression as an H3K4-specific demethylase directly targeting *FT* locus with the following data: 1) The loss of *AtJmj4* function increased *FT* expression via enhanced *FT* promoter activity (**Figure 2**); 2) The loss of *AtJmj4* function increased H3K4me3 level in the transcription initiation region of *FT* (**Figures 6C** and **6D**); 3) The purified AtJmj4 protein can specifically demethylate H3K4me1, H3K4me2, and H3K4me3 *in vitro* (**Figure 6E**); 4) The AtJmj4::FLAG binds to the transcription initiation region of *FT*. In addition, up-regulation of *FT* expression could also be responsible, at least in part, for the increased H3K4me3 level in *atjmj4* at the *FT* locus because chromatin modification often involves positive feedback loops [44].

It is notable that the increase of H3K4me3 at *FT* caused by the loss *AtJmj4* is modest. This indicated that there might be other histone demethylases having redundant roles with AtJmj4 in the demethylation of H3K4 at the *FT* locus. Indeed, we found that ELF6 is another histone demethylase with similar activity towards *FT* based on the following data: 1) The loss of *ELF6* function increased *FT* expression and the H3K4me3 level in the transcription initiation region of *FT*, and these increases were more significant when *AtJmj4* and *ELF6* functions were lost together (**Figures 5D**, **5E**, **6C**, and **6D**); 2) ELF6::GUS binds to the promoter and transcription initiation region of *FT*. Previously we reported that both AtJmj4 and ELF6 belong to the same group (Group I) of Arabidopsis Jmj family proteins [21]. Our unpublished phylogenetic analysis indicates that eight Arabidopsis Jmj family proteins belonging to this group have JmjC domains clustered together with the JmjC domains of human JARID1 family that are H3K4 demethylases [17], [19]. Therefore, not only AtJmj4 and ELF6 but also other members of the Arabidopsis Group I Jmj family proteins have a potential to be H3K4 demethylases acting at *FT* locus. Their genetic and biochemical roles will be addressed in the future studies.

According to recent studies, the antagonistic histone marks, H3K27me3 and H3K4me3, are coordinately regulated by protein complexes containing both histone methyltransferases and histone demethylases [48], [49]. Pasini et al [49] reported that the RBP2 H3K4 demethylase is recruited by the PRC2 to repress the expression of target genes in mouse embryonic stem cells, and the loss of RBP2 increases expression of target genes. At this moment, it is not clear if a similar interaction between AtJmj4/ELF6 and the Arabidopsis PRC2 components occurs in *FT* repression. However, the report that the level of H3K4me3 within *FT* chromatin is increased in the absence of CURLY LEAF activity [12] suggests such scenario is plausible. In this study, we did not observe a significant reduction of H3K27me3 level within *FT* chromatin in *elf6 atjmj4* double mutants. Thus, H3K4 demethylases might be recruited by PRC2, but the PRC2 recruitment might not be affected by H3K4 demethylases.

The coexistence of bivalent H3K27me3 and H3K4me3 marks at the same locus has been proposed to poise genes for activation upon appropriate developmental cues [50], [51]. Thus, the existence of bivalent chromatin marks within *FT* chromatin might be a strategy for plants to achieve reproductive success by a precise regulation of *FT* expression and flowering time. It is possible that enriched H3K27me3 favors constitutive *FT* repression, while a proper level of H3K4me3 provides appropriate accessibility for transcription factors controlled temporally such that *FT* expression can be regulated by changing developmental or environmental cues. Interestingly, the region I of *FT* locus, in which both AtJmj4::FLAG and ELF6::GUS showed strongest binding (**Figures 7A–7C**) and the largest increase of H3K4me3 by the *elf6* and *atjmj4* mutations was observed (**Figures 6A**, **6C**, and **6D**), contains binding sites for *FT* transcriptional regulators, namely TEM1/TEM2 [39] and a CO-containing protein complex [52]. Thus, it would be of interest in the future to test if the binding of these *FT* transcriptional regulators is altered by the activity of AtJmj4 and ELF6.

## Materials and Methods

### Plant Materials and Growth

*atjmj4* T-DNA insertion lines in the Col background were obtained from the SALK collection (http://signal.salk.edu/; *atjmj4-1*, SALK_135712; *atjmj4-2*, SALK_136058). The following mutants are in the Col background and were described previously: *elf6-4* [22], *vin3-5* [53], *flc-3* [29], *gi-2* [1], *co-101* [14], *ft-10* [54], *lhp1-4* [55], *fld-3* [31], *fve-4* [32], *FRI* [33]. All plants were grown under 100 µE m^−2^ s^−1^ cool white fluorescent light at 22°C.

### T-DNA Flanking Sequence Analysis

The T-DNA borders of *atjmj4-1* and *atjmj4-2* alleles were defined by sequencing PCR products obtained using a T-DNA border primer (SALKLB1; Table S1) and gene-specific primers. For *atjmj4-1*, AtJmj4-1-R and AtJmj4-1-F primer pair (Table S1) was used to detect wt allele while AtJmj4-1-R and SALKLB1 primer pair was used to detect *atjmj4-1* allele. For *atjmj4-2*, AtJmj4-2-F and AtJmj4-2-R primer pair (Table S1) was used to detect wt allele while AtJmj4-2-F and SALKLB1 primer pair was used to detect *atjmj4-2* allele.

### RT-PCR and qPCR Analyses

Total RNA was isolated from seedlings using TRI Reagent (Molecular Research Center, INC.) according to the manufacturer's instructions. RT was performed with M-MuLV Reverse Transcriptase (Fermentas) according to the manufacturer's instructions using 3 µg of total RNA. PCR was performed on first strand DNA with i-Taq DNA polymerase (iNtRON Biotechnology). Primers used for RT-PCR and qPCR analyses are listed in Table S2. qPCR was performed in 96-well blocks with an Applied Biosystems 7300 real-time PCR system using the SYBR Green I master mix (Bio-Rad) in a volume of 20 µl. The reactions were performed in triplicate for each run. The comparative _ΔΔ_CT method was used to evaluate the relative quantities of each amplified product in the samples. The threshold cycle (Ct) was automatically determined for each reaction by the system set with default parameters.

### GUS and GFP Assays

The *FT_pro_::GUS* [54] and the *ELF6::GUS* [22] were described previously. For the construction of the *AtJmj4::GUS* translational fusion construct, a 6-kb genomic DNA fragment of *AtJmj4* containing 1.5-kb 5′ upstream region and the entire coding region was generated by PCR amplification using AtJmj4GUS-F and AtJmj4GUS-R as primers (Table S3). After restriction digestion with *Sal*I-*Sma*I, the PCR product was ligated into pPZP211-GUS [56] at *Sal*I-*Sma*I sites. The final construct was introduced into wt Col plants by the floral dip method [57] through *Agrobacterium tumefaciens* strain C58C1, and transformants were selected on MS media supplemented with 1% sucrose and 50 µg ml^−1^ kanamycin. Histochemical GUS staining was performed as described [58].

*AtJmj4* cDNA obtained from Col RNA through RT-PCR using AtJmj4OE-F and AtJmj4OE-R as primers (Table S3) was used for the construction of the *CaMV35S_pro_::AtJmj4::GFP*. The cDNA was cloned into the *Sal*I site of p35SsGFP/pGEM which has the open reading frame of *sGFP* [59] behind the Cauliflower Mosaic Virus 35S promoter (*CaMV35S_pro_*). Mesophyll protoplasts were isolated from rosette leaves of Col plants grown for 4 weeks in LD as described [60]. The LHP1::RFP [43] was included as a nuclear protein control. Protoplasts were co-transformed with the *CaMV35S_pro_::AtJmj4::GFP* and the *LHP1::RFP* constructs, each with 10 µg of plasmid DNA prepared with Nucleo Bond Xtra Midi Kit (Macherey-Nagel). After 16 h incubation at 22°C in dark, protoplasts were observed with LSM 510 confocal microscope (Zeiss). The GFP and RFP fusion proteins were excited at 488 nm and 543 nm, respectively. The autofluorescence of chlorophylls, GFP, and RFP were analyzed with LP650, BP500-530IR, and BP565-615IR filters, respectively. The merged image was obtained using the LSM Image Browser (Zeiss).

### AtJmj4::FLAG

The *AtJmj4::FLAG* construct is consisted of a 0.8 kb 5′ upstream region of *AtJmj4* (*AtJmj4_pro_*), the sequence for 3xFLAG tags, and the full coding sequence of *AtJmj4* cDNA. *AtJmj4* cDNA was obtained from Col RNA through RT-PCR using AtJmj4OE-F and AtJmj4OE-R1 as primers (Table S3), digested with *Sal*I, and cloned into the *Sal*I site of a construct containing *3xFLAG* behind the *CaMV35S_pro_* in pPZP211 vector. The *CaMV35S_pro_* was replaced with the *AtJmj4_pro_* obtained from Col genomic DNA through PCR using Atjmj4FLAG-F and Atjmj4FLAG-R as primers (Table S3) at *Pst*I site. Then the *AtJmj4_pro_::3xFLAG::AtJmj4 cDNA* was PCR-amplified using AtJmj4FLAG-F1 and Atjmj4FLAG-R1 as primers (Table S3). After restriction digestion with *Nhe*I, the PCR fragment was ligated into the *Sma*I-*Xba*I sites of the binary vector pPZP221B [61]. The final construct was introduced into *atjmj4-1* mutants by the floral dip method through *Agrobacterium tumefaciens* strain C58C1, and transformants were selected on MS media supplemented with 1% sucrose and 25 µg ml^−1^ glufosinate ammonium.

Protein samples were extracted using 2xloading buffer from wt Col and transgenic plants harboring the *AtJmj4::FLAG* construct, and their concentrations were determined by Protein Assay (Bio-Rad). 3.75 µg of protein samples were size-fractionated on a 7% SDS-PAGE gel, transferred to Pure Nitrocellulose (GE Water & Process Technologies), and blocked with 10% skim milk power in TTBS (0.1% tween 20, 20 mM Tris-HCl pH7.4, 150 mM NaCl). AtJmj4::FLAG protein was detected using Anti-FLAG M2-Peroxidase (HRP) antibody (Sigma), ECL Western Blotting Detection Kit (GE Healthcare), and JP/LAS-3000 Luminescent Image Analyzer (Fujifilm).

### AtJmj4 Protein Expression and Purification

For the expression of AtJmj4 protein in insect cells, the full length *AtJmj4* coding region was PCR amplified from a cDNA clone using JMJ4_pENTR_For and JMJ4_pENTR_Rev as primers (Table S3) and ligated into the Klenow-filled *EcoR*I site of pFastBac HT A vector (Invitrogen). The resulting *AtJmj4::pFastBac HT A* construct with amino-terminal 6xHis tag was used to transform DH10Bac *E. coli* competent cells (Invitrogen), and the recombinant baculovirus DNA was selected and used for the infection of *sf9* cells following the Bac-to-Bac system instructions (Invitrogen). Cells positive for the recombinant-protein expression as tested by western blot with anti-His antibody (Santa Cruz) was used to infect cells to produce *6His-AtJmj4* baculovirus stocks. Viral stocks were stored at 4°C. For protein expression, 2.5 ml of viral stock was used to infect approximately 2×10^6^ adherent *sf9* cells in 400 ml of sf-900 II SFM serum free medium (Gibco) and cultured at 27°C for 48 h. Then, cells were harvested and washed with PBS and frozen at −80°C until further purification. Frozen cells were thawed on ice and resuspended with 20 ml equilibration buffer (80 mM Na_2_HP0_4_, 20 mM NaH_2_PO_4_, 300 mM NaCl, 10 mM imidazole, 100 µM PMSF, 10% glycerol). Cells were disrupted by sonication, and the lysate was clarified by centrifugation at 10,000× g for 20 min at 4°C. The supernatant was applied into a Ni-NTA-Agarose (Qiagen) chromatography column and washed with 10× column volume of equilibration buffer. Protein was eluted from the column with 3× bed volume of elution buffer (80 mM Na_2_HP0_4_, 20 mM NaH_2_PO_4_, 300 mM NaCl, 250 mM imidazole, 100 µM PMSF, 10% glycerol). Purified recombinant 6His-AtJmj4 protein was dialyzed against dialysis buffer (40 mM HEPES-KOH pH 7.9, 50 mM KCl, 10% glycerol, 1 mM DTT, 0.2 mM PMSF) overnight at 4°C. Next-day, dialyzed 6His-AtJmj4 proteins was quantified and stored at −20°C.

### Histone Demethylase Assay

*In vitro* histone demethylation assay was performed as previously described [62] with minor modifications. Briefly, two or four µg of purified 6His-AtJmj4 protein was incubated with 4 µg of calf thymus histones type II-A (Sigma) in the DeMTase reaction buffer 1 (20 mM Tris-HCl pH 7.3, 150 mM NaCl, 50 mM (NH_4_)_2_Fe(SO_4_)_2_·6(H_2_O), 1 mM α-ketoglutarate, 2 mM ascorbic acid) for 5 h at 37°C. Histone modifications were detected by western blot analysis with antibodies as follows: anti-H3K4me1 (Upstate 07-436), anti-H3K4me2 (Upstate 07-030), anti-H3K4me3, (Abcam ab8580), anti-H3K9me3 (Upstate 07-442), anti-H3K36me2 (Upstate 07-369), anti-H3K27me3 (Upstate 07-449), and anti-H3 (Abcam ab1791-100).

### ChIP Assay

ChIP was performed as described by Han et al. [63] using 55- to 60-d-old plants grown in SD. Briefly, leaves were vacuum infiltrated with 1% formaldehyde for cross-linking and ground in liquid nitrogen after quenching the cross-linking process. Chromatin was isolated and sonicated into ∼0.5 to 1 kb fragments. Specific antibody against GUS (Invitrogen A5790), FLAG (Sigma A8592-0.2MG), H3K4me3 (Upstate 07-473), or H3K27me3 (Upstate 07-449) was added to the chromatin solution, which had been precleared with salmon sperm DNA/Protein A agarose beads (Upstate 16-157). After subsequent incubation with salmon sperm DNA/Protein A agarose beads, immunocomplexes were precipitated and eluted from the beads. Cross-links were reversed, and residual proteins in the immunocomplexes were removed by incubation with proteinase K, followed by phenol/chloroform extraction. DNA was recovered by ethanol precipitation. The amount of immunoprecipitated *FT*, *CO*, and *Actin1* chromatins was determined by PCR with primer pairs in Table S4.

## Supporting Information

Figure S1 Leaf initiation rate of atjmj4-1 mutants: Wt Col (black circles) and atjmj4-1 mutant plants (white squares) were grown in SD and their leaf numbers were scored every week from four weeks after planting. At least 10 individuals were scored for each genotype. Error bars represent sd.
(0.13 MB TIF)

Figure S2 FLC-independent function of AtJmj4: Expression of flowering genes in flc-3 and atjmj4-1 mutant plants grown in SD for 12 d as determined by RT-PCR analysis. UBQ was used as an expression control.
(0.25 MB TIF)

Figure S3 Vernalization response of atjmj4 mutants: Plants of each genotype were treated with vernalization for 40 d as described previously [56]. Flowering time was scored as leaf number for plants either without (V0) or after (V40) vernalization treatment. At least 12 individuals were scored for each genotype. Error bars represent sd.
(0.36 MB TIF)

Figure S4 CO- and GI-independent increase of FT expression in atjmj4: Plants of each genotype were grown in LD for 14 d and harvested at ZT8 for RT-PCR analyses. UBQ was used as an expression control.
(0.22 MB TIF)

Figure S5 Expression of FT regulators in atjmj4: A and B) Temporal expression of FT regulators in atjmj4-1. Col and atjmj4-1 plants were grown in SD (A) or in LD (B) until indicated DAG and harvested at ZT14 (LD) or ZT8 (SD) for RT-PCR analyses. UBQ was used as an expression control.
(0.60 MB TIF)

Table S1 Oligonucleotides used for T-DNA flanking sequence analysis
(0.03 MB DOC)

Table S2 Oligonucleotides used for RT-PCR analysis
(0.05 MB DOC)

Table S3 Oligonucleotides used for constructs
(0.03 MB DOC)

Table S4 Oligonucleotides used for ChIP assay
(0.04 MB DOC)
