# Supplementary figures and images for: Repression of FLOWERING LOCUS T Chromatin by Functionally Redundant Histone H3 Lysine 4 Demethylases in Arabidopsis
Source: PLoS One. 2009 Nov 25;4(11):e8033. doi: 10.1371/journal.pone.0008033 (PMC2777508; doi:10.1371/journal.pone.0008033)

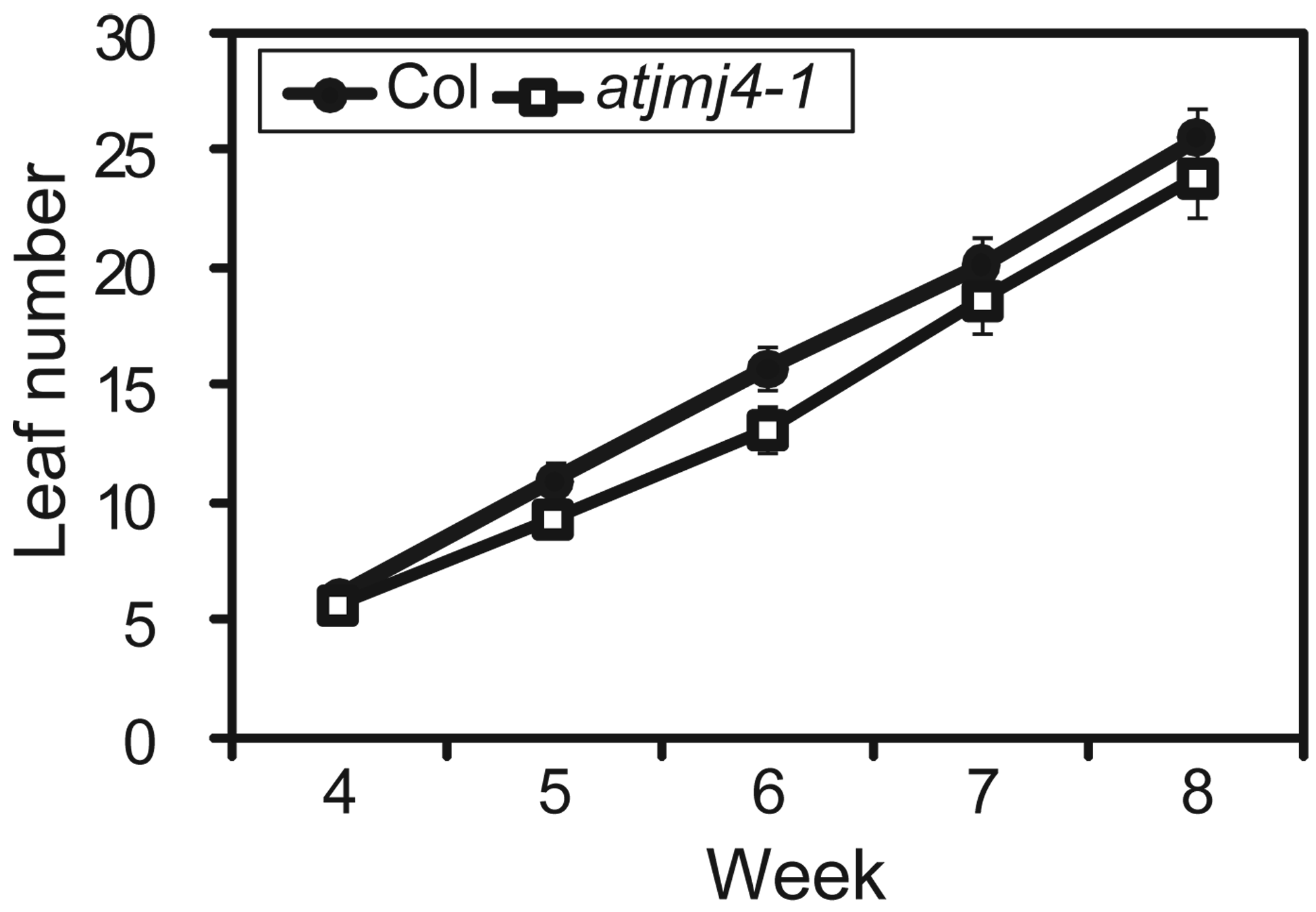

Supplement: Figure S1 — Leaf initiation rate of atjmj4-1 mutants: Wt Col (black circles) and atjmj4-1 mutant plants (white squares) were grown in SD and their leaf numbers were scored every week from four weeks after planting. At least 10 individuals were scored for each genotype. Error bars represent sd. (0.13 MB TIF) [file pone.0008033.s001.tif]

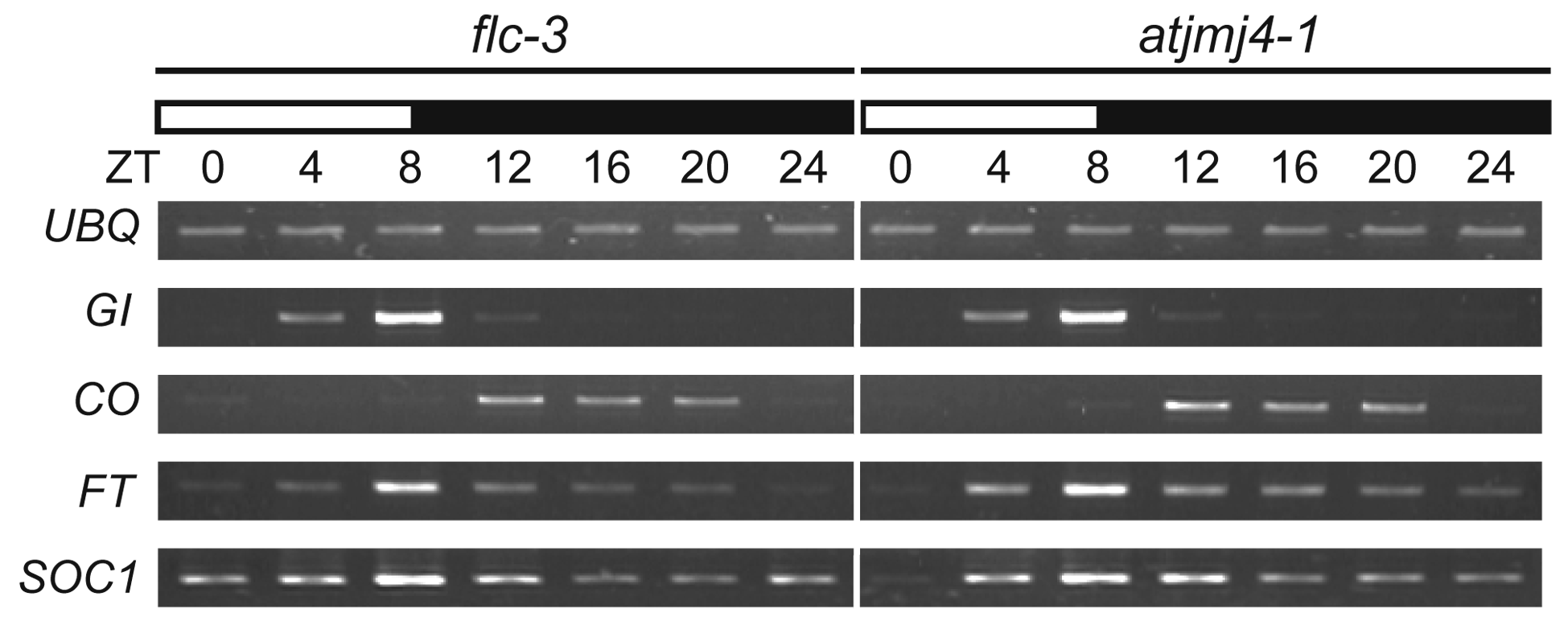

Supplement: Figure S2 — FLC-independent function of AtJmj4: Expression of flowering genes in flc-3 and atjmj4-1 mutant plants grown in SD for 12 d as determined by RT-PCR analysis. UBQ was used as an expression control. (0.25 MB TIF) [file pone.0008033.s002.tif]

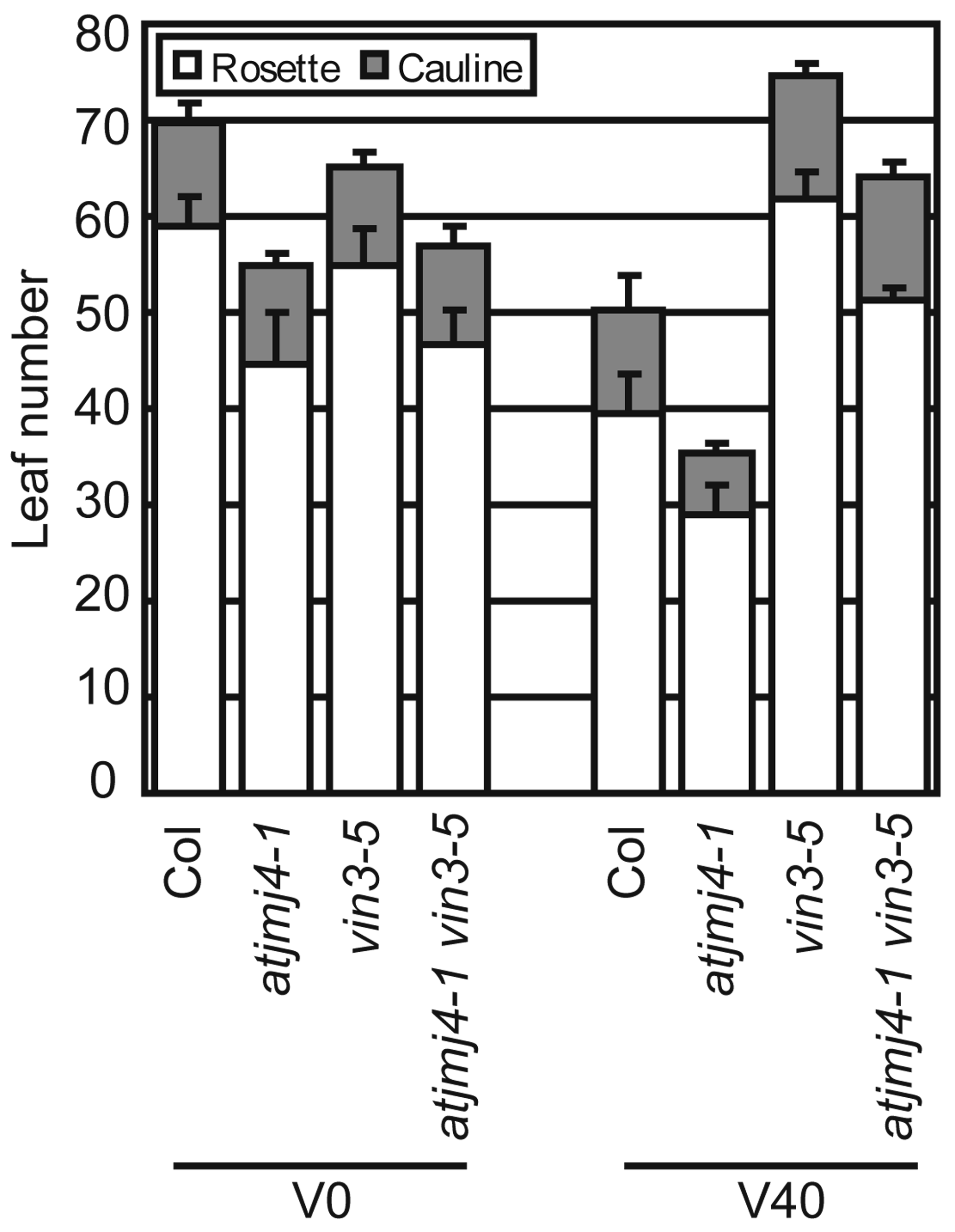

Supplement: Figure S3 — Vernalization response of atjmj4 mutants: Plants of each genotype were treated with vernalization for 40 d as described previously [56]. Flowering time was scored as leaf number for plants either without (V0) or after (V40) vernalization treatment. At least 12 individuals were scored for each genotype. Error bars represent sd. (0.36 MB TIF) [file pone.0008033.s003.tif]

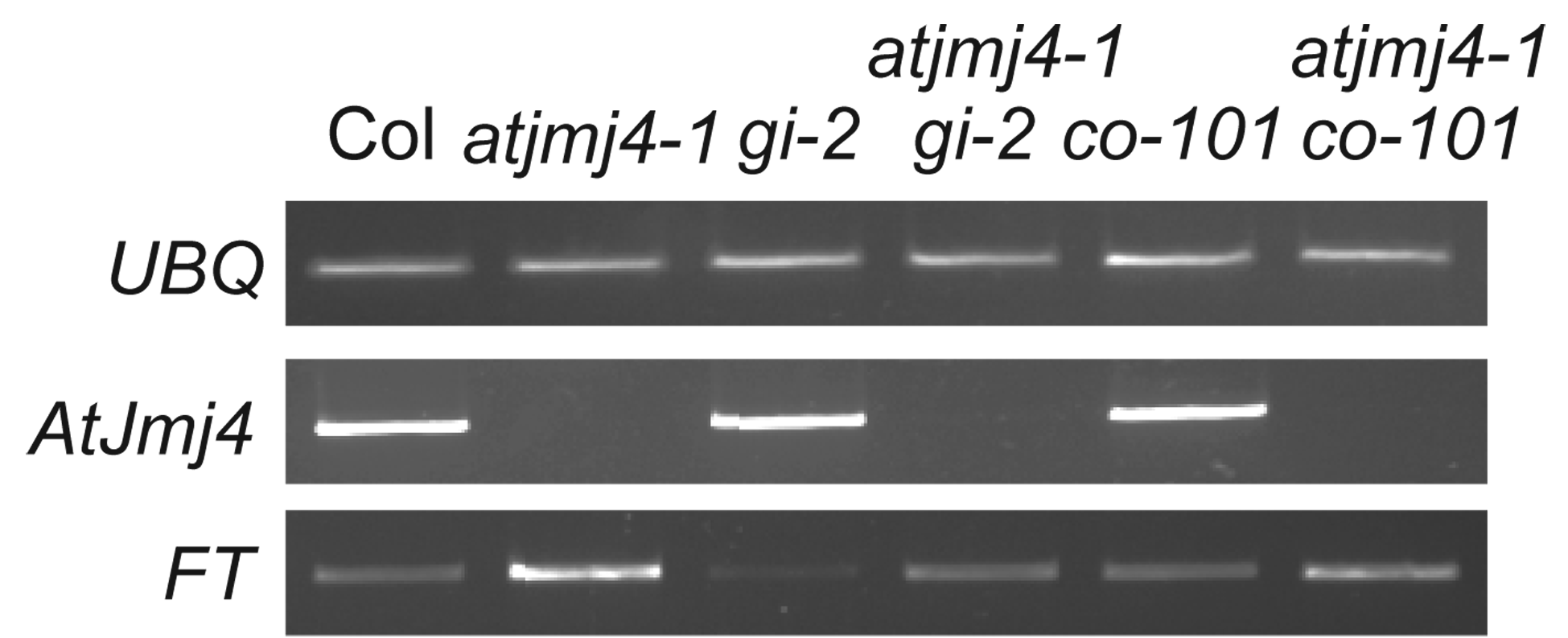

Supplement: Figure S4 — CO- and GI-independent increase of FT expression in atjmj4: Plants of each genotype were grown in LD for 14 d and harvested at ZT8 for RT-PCR analyses. UBQ was used as an expression control. (0.22 MB TIF) [file pone.0008033.s004.tif]

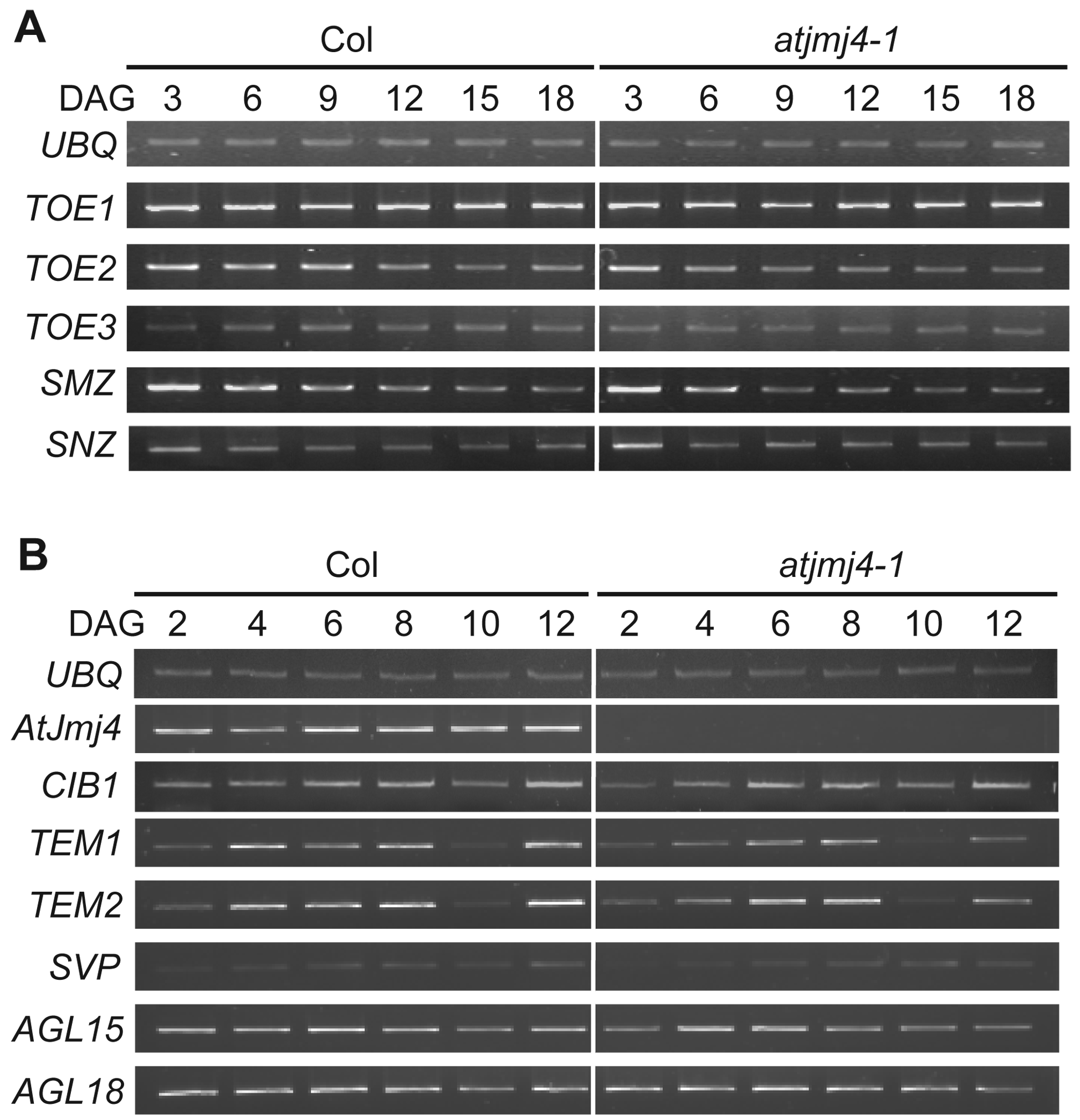

Supplement: Figure S5 — Expression of FT regulators in atjmj4: A and B) Temporal expression of FT regulators in atjmj4-1. Col and atjmj4-1 plants were grown in SD (A) or in LD (B) until indicated DAG and harvested at ZT14 (LD) or ZT8 (SD) for RT-PCR analyses. UBQ was used as an expression control. (0.60 MB TIF) [file pone.0008033.s005.tif]
