# Supplementary material for: Repression of FLOWERING LOCUS T Chromatin by Functionally Redundant Histone H3 Lysine 4 Demethylases in Arabidopsis
Source: PLoS One. 2009 Nov 25;4(11):e8033. doi: 10.1371/journal.pone.0008033 (PMC2777508; doi:10.1371/journal.pone.0008033)
Supplement: Table S1 — Oligonucleotides used for T-DNA flanking sequence analysis (0.03 MB DOC) [file pone.0008033.s006.doc]

**Table S1 Oligonucleotides used for T-DNA flanking sequence analysis**

| Name | Sequence |
| --- | --- |
| SALKLB1 | 5’-GCAAACCAGCGTGGACCGCTTGCTGCAACT-3’ |
| AtJmj4-1-F | 5’-GAGAAGTTGCGCTCTAAAGCAGAATC-3’ |
| AtJmj4-1-R | 5’-TGGATCACCTGTGTGTAAGTAGTTCATGG -3’ |
| AtJmj4-2-F | 5’-AATATGTATCTCACTCTGCACC-3’ |
| AtJmj4-2-R | 5’-AAATCACCCTCCATCTTTCGC-3’ |
