# Supplementary material for: Repression of FLOWERING LOCUS T Chromatin by Functionally Redundant Histone H3 Lysine 4 Demethylases in Arabidopsis
Source: PLoS One. 2009 Nov 25;4(11):e8033. doi: 10.1371/journal.pone.0008033 (PMC2777508; doi:10.1371/journal.pone.0008033)
Supplement: Table S2 — Oligonucleotides used for RT-PCR analysis (0.05 MB DOC) [file pone.0008033.s007.doc]

**Table S2** Oligonucleotides used for RT-PCR analysis

| Gene | Name | Sequence |
| --- | --- | --- |
| *Ubiquitin* | UBQ-F | 5’-GATCTTTGCCGGAAAACAATTGGAGGATGGT-3’ |
|  | UBQ-R | 5’-CGACTTGTCATTAGAAAGAAAGAGATAACAGG-3’ |
| *AtJmj4* | AtJmj4-F | 5’-GCTTGACCCAACAAACCTAACC-3’ |
|  | AtJmj4-R | 5’-TCTCACCACACAGAAGTCCATGC-3’ |
| *GI* | GI-F | 5’-GTTGTCCTTC AGGCTGAAAG-3’ |
|  | GI-R | 5’-TGTGGAGAGC AAGCTGTGAG-3’ |
| *CO* | CO-F | 5’-AAACTCTTTCAGCTCCATGACCACTACT-3’ |
|  | CO-R | 5’-CCATGGATGAAATGTATGCGTTATGGTTA-3’ |
| *FT* | FT-F | 5’-GCTACAACTGGAACAACCTTTGGCAAT-3’ |
|  | FT-R | 5’-TATAGGCATCATCACCGTTCGTTACTC-3’ |
| *SOC1* | SOC1-F | 5’-TGAGGCATACTAAGGATCGAGTCAG-3’ |
|  | SOC1-R | 5’-GCGTCTCTACTTCAGAACTTGGGC-3’ |
| *FLC* | FLC-F | 5’-TTCTCCAAACGTCGCAACGGTCTC-3’ |
|  | FLC-R | 5’-GATTTGTCCAGCAGGTGACATCTC-3’ |
| *TOE1* | TOE1-F | 5’-ACTCAGTACGGTGGTGACTC-3’ |
|  | TOE1-R | 5’-CGAGGATCCATAAGGAAGAGG-3’ |
| *TOE2* | TOE2-F | 5’-CACTTTCTATCGGAGGACAG-3’ |
|  | TOE2-R | 5’-CTTCCACATACGGAATTGTT-3’ |
| *TOE3* | TOE3-F | 5’-GTTACGTTTTACCGACGAAC-3’ |
|  | TOE3-R | 5’-TGCTTGCAATATCAGACTTG-3’ |
| *SMZ* | SMZ-F | 5’-AATGGTGAAGAAGAGCAGAA-3’ |
|  | SMZ-R | 5’-CTTTCCGATGATGATGAAAT-3’ |
| *SNZ* | SNZ-F | 5’-TTTGGAATCCTTAAACGAAA-3’ |
|  | SNZ-R | 5’-TATCTCATTGCATTTTGCTG-3’ |
| *CIB1* | CIB1-F | 5’-GCATAGCAGAACGAGTTAGAAGAG-3’ |
|  | CIB1-R | 5’-ATCAGAACTGGTATTCACTTGCTG-3’ |
| *TEM1* | TEM1-F | 5’-GCGTGTTGTTTCGGTATCACTA-3’ |
|  | TEM1-R | 5’-ATTCAGAGAACGGCGTCGA-3’ |
| *TEM2* | TEM2-F | 5’-TTCCTCAGCCTAACGGAAGAT-3’ |
|  | TEM2-R | 5’-TCCTTGACGAATCGACTCCAT-3’ |
| *SVP* | SVP-F | 5’-CGCTCTCATCATCTTCTCTTCCAC-3’ |
|  | SVP-R | 5’-GCTCGTTCTCTTCCGTTAGTTGC-3’ |
| *AGL15* | AGL15-F | 5’-TTATCTAGATGGGTCGTGGAAAAATCGAG-3’ |
|  | AGL15-R | 5’-TTAGCGGCCGCAGAGAACCTTTGTCTTTTGGCTTC-3’ |
| *AGL18* | AGL18-F | 5’-ATGGGGAGAGGAAGGATTGAGATTAAGAA-3’ |
|  | AGL18-R | 5’-TCAATCAGAAGCCACTTGACTCCCAGAGT-3’ |
