# Supplementary material for: Repression of FLOWERING LOCUS T Chromatin by Functionally Redundant Histone H3 Lysine 4 Demethylases in Arabidopsis
Source: PLoS One. 2009 Nov 25;4(11):e8033. doi: 10.1371/journal.pone.0008033 (PMC2777508; doi:10.1371/journal.pone.0008033)
Supplement: Table S3 — Oligonucleotides used for constructs (0.03 MB DOC) [file pone.0008033.s008.doc]

**Table S3** Oligonucleotides used for constructs

| Name | Sequence |
| --- | --- |
| AtJmJ4GUS-F | 5’-gtcgacGTCTCCTCTCTATCGCCATTCTTG-3’ |
| AtJmJ4GUS-R | 5’-CTTcccgggAAAGGACTTATCTCCATC-3’ |
| AtJmJ4OE-F | 5’-CAgtcgacATGGATCAGCTTGCATCTC-3’ |
| AtJmJ4OE-R | 5’-TGCgtcgacAAGGACTTATCTCCATC-3’ |
| AtJmJ4OE-R1 | 5’-TGCgtcgacTTAAGGACTTATCTCCATC-3’ |
| AtJmj4FLAG-F | 5’-TTctgcagGTCTTCGTCTCCTCTCTATCGC-3’ |
| AtJmj4FLAG-R | 5’-GGGctgcagCATTTACAGTGAGATTAAGTTC-3’ |
| AtJmj4FLAG-F1 | 5’-GAgctagcTCCTCTCTATCGCCATTCTT-3’ |
| AtJmj4FLAG-R1 | 5’-GTCTCGAGGAATTCCCAAACATATAGTAGATG-3’ |
| JMJ4_pENTR_For | 5’-CACCATGGATCAGCTTGCATCTCTAG-3’ |
| JMJ4_pENTR_Rev | 5’-AGGACTTATCTCCATCTTATC-3’ |

Restriction sites used for cloning are in small letters and underlined**.**
