# Supplementary material for: Repression of FLOWERING LOCUS T Chromatin by Functionally Redundant Histone H3 Lysine 4 Demethylases in Arabidopsis
Source: PLoS One. 2009 Nov 25;4(11):e8033. doi: 10.1371/journal.pone.0008033 (PMC2777508; doi:10.1371/journal.pone.0008033)
Supplement: Table S4 — Oligonucleotides used for ChIP assay (0.04 MB DOC) [file pone.0008033.s009.doc]

**Table S4** Oligonucleotides used for ChIP assay

| Gene | Region | Name | Sequence |
| --- | --- | --- | --- |
| *FT* | F | FF | 5’-ACTTGGCGGTACCCTACTT-3’ |
|  |  | FR | 5’-ATATCTCCCACTTGGTAG -3’ |
|  | G | GF | 5’-GTCGAGAGAGGTATCTTGTTAAAG-3’ |
|  |  | GF | 5’-ATCATAGGCATGAACCCTCTACAC-3’ |
|  | I | IF | 5’-TATGTGTAGAGGGTTCATGCCTATG-3’ |
|  |  | IR | 5’-TGGCCATAACCTTTAGAGTG -3’ |
|  | Ia | IaF | 5’-CCACCTGTTTGTTCAAGATC-3’ |
|  |  | IaR | 5’-GAAGGCCTTAGATCCAAGCC-3’ |
|  | EX1 | EX1F | 5’-ATGTCTATAAATATAAGAGACCCTC-3’ |
|  |  | EX1R | 5’-CTTCTCCACCAATCTCAACTCTTG-3’ |
|  | N | NF | 5’-TCCACCAACTTCTTGCATAAGTGA-3’ |
|  |  | NR | 5’-CCACAACAGAGATTCATCAATATAT-3’ |
| *Actin1* |  | Actin1 F | 5’-CGTTTCGCTTTCCTTAGTGTTAGCT-3’ |
|  |  | Actin1 R | 5’- AGCGAACGGATCTAGAGACTCACCTTG-3’ |
| *CO* |  | CO F | 5’-GGCACTCAGGATTCGATCTCC-3’ |
|  |  | CO R | 5’-CCGGCATGTGTCACAGGGTCG-3’ |
